# Phytoecdysteroids from the Roots of *Achyranthes bidentata* Blume

**DOI:** 10.3390/molecules17033324

**Published:** 2012-03-14

**Authors:** Mei Zhang, Zhong-Yu Zhou, Jing Wang, Yong Cao, Xue-Xiang Chen, Wei-Min Zhang, Li-Dong Lin, Jian-Wen Tan

**Affiliations:** 1 Key Laboratory of Plant Resources Conservation and Sustainable Utilization, South China Botanical Garden, Chinese Academy of Sciences, Guangzhou 510650, China; 2 Graduate School of the Chinese Academy of Sciences, Beijing 100039, China; 3 College of Food Science, South China Agricultural University, Guangzhou 510642, China; 4 Guangdong Provincial Key Laboratory of Microbial Culture Collection and Application, Guangdong Institute of Microbiology, Guangzhou 510070, China

**Keywords:** *Achyranthes bidentata*, Amaranthaceae, phytoecdysteroids, ecdysteroids

## Abstract

Two new phytoecdysteroids, (25*S*)-20,22-*O*-(*R*-ethylidene)inokosterone (**1**) and 20,22-*O*-(*R*-3-methoxycarbonyl)propylidene-20-hydroxyecdysone (**2**), together with six known phytoecdysteroids **3**–**8** were isolated from the roots of *Achyranthes bidentata* Blume. The new structures were established on the basis of spectroscopic studies and chemical evidences. The absolute configuration at C-25 in the structure of known compound **3** was determined by chemical and spectroscopic means.

## 1. Introduction

**Figure 1:**
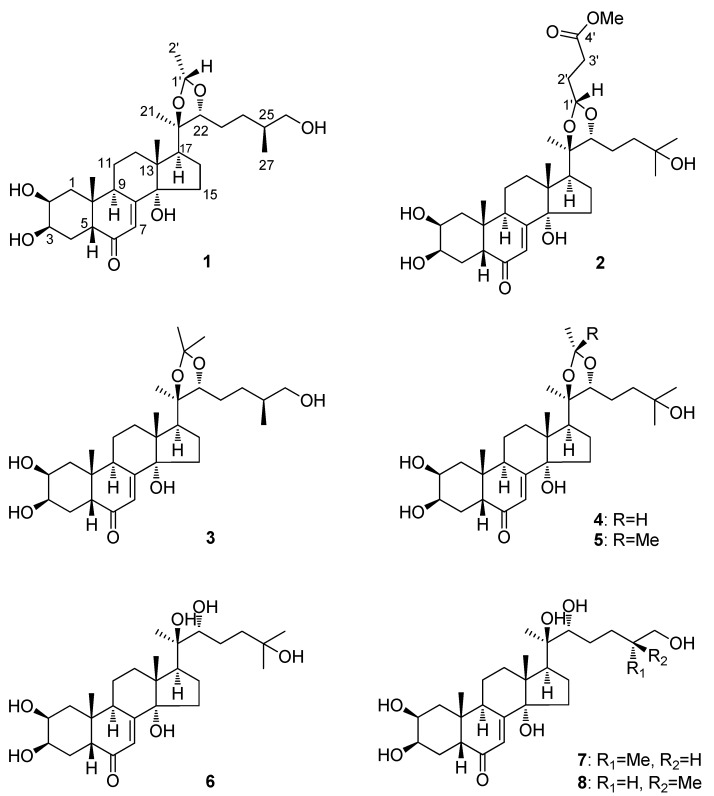
Chemical structures of compounds **1**–**8***.*

*Achyranthes bidentata* Blume (Amaranthaceae) is widely distributed in Asian countries like India, Korea, Japan, and China. The root of *A. bidentata* has been prescribed in the Chinese Pharmacopeia as an important herbal medicine and its multiple pharmacological effects, such as anti-osteoporosis [1,2], antitumor [3,4,5], anti-senility [3,6,7,8], anti-inflammatory [9], immunomodulatory [3,10,11,12,13] activities are well documented. Previous phytochemical investigations of *A. bidentata* have reported the isolation of phytoecdysteroids [14,15,16], saccharides [17,18] and saponins [19,20], and some of them displayed diverse bioactivities. In the course of our search for potentially new and bioactive compounds from medicinal plants in China, we investigated the roots of *A. bidentata* and isolated eight phytoecdysteroids, including two new ones, (25*S*)-20,22-*O*-(*R*-ethylidene)inokosterone (**1**) and 20,22-*O*-(*R*-3-methoxycarbonyl)propylidene-20-hydroxyecdysone (**2**), and six known phytoecdysteroids **3–8** (Figure 1). In this paper, we report the isolation and structural elucidation of these phytoecdysteroids.

## 2. Results and Discussion

**Figure 2:**
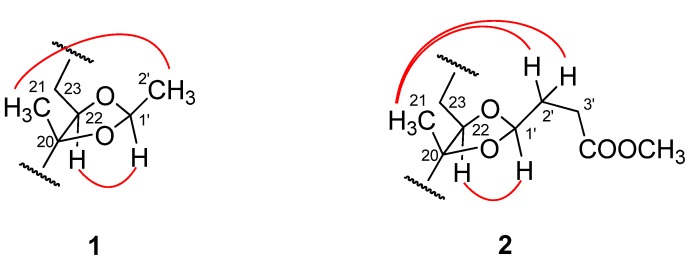
Key NOE (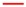) correlations of **1** and **2**.

Compound **1** was obtained as a white amorphous powder with a molecular formula of C_29_H_46_O_7_ as established on the basis of a combined analysis of its HRESIMS and NMR spectroscopic data. The spectroscopic data (IR, UV, and NMR: see Section 3.3. and Table 1 and Table 2) supported the fact that **1** was an ecdysteroid closely related to inokosterone-20,22-acetonide, a reported [21,22] ecdysteroid compound which was also obtained as compound **3** in the present study. Careful analysis of the ^1^H- and ^13^C-NMR spectra pointed out as major differences that the resonances for the acetone ketal group in **3** were replaced by the signals for a C_2_ acetal group [*δ*_H_ 5.05 (1H, q, H-1'), 1.29 (3H, d, H_3_-2'); *δ*_C_ 102.3 (C-1'), 22.0 (C-2')]. These data led us to preliminarily establish the whole structure of **1** as 20,22-ethylideneinokosterone, of which the stereochemistry at C-25 and C-1' still needed to be determined. In the ^1^H-^1^H COSY spectrum, signals correlated to the connections of C-22 to C-27 were exhibited. In the NOESY spectrum, a significant correlation signal of H-1' (*δ*_H_ 5.05) with H-22 (*δ*_H_ 3.65) was observed, which indicated that the stereochemistry at C-1' in **1** should be *R* configuration (Figure 2). In order to clarify the absolute configuration at C-25 in the molecule, an acid hydrolysis of **1** was carried out, and the centre ecdysteroid unit that released from **1** was confirmed to be the same as the known compound (25*S*)-inokosterone (**8**), as evidenced by a co-TLC elution test and comparison of the NMR spectroscopic data with literature precedents [23]. This result indicated that the absolute configuration at C-25 of **1** should be *S*. Thus, the complete structure of **1** was elucidated as (25*S*)-20,22-*O*-(*R*-ethylidene)inokosterone.

**Table 1:** The ^1^H-NMR (400 MHz) spectral data [*δ* (ppm), *J* in Hz] of **1**–**4** and **8**.

No.	*δ*_H_ (1) ^a^	*δ*_H_ (2) ^a^	*δ*_H_ (3) ^a^	*δ*_H_ (4) ^a^	*δ*_H_ (8) ^b^
1	1.77 (m), 1.41(m)	1.78 (m)	1.78 (m)	1.79(m)	2.15 (m), 1.93 (m)
2	3.82 (m)	3.82 (m)	3.82 (m)	3.82 (m)	4.19 (m)
3	3.94 (m)	3.94 (m)	3.94 (m)	3.94 (m)	4.23 (m)
4	1.73 (m), 1.69 (m)	1.71 (m)	1.73 (m), 1.69 (m)	1.72 (m)	2.04 (m), 1.82 (m)
5	2.38 (m)	2.36 (m)	2.37 (m)	2.35 (m)	3.01 (dd, 13.2, 3.2)
7	5.80 (d, 2.0)	5.81 (s)	5.80 brs	5.80 (d, 2.0)	6.26 (d, 1.6)
9	3.13 (m)	3.13 (m)	3.13 (m)	3.13 (m)	3.60 (m)
11	1.79 (m), 1.67 (m)	1.79 (m), 1.68 (m)	1.79 (m), 1.67 (m)	1.78 (m), 1.68 (m)	1.88 (m), 1.73(m)
12	2.09 (m), 1.82 (m)	2.09 (m), 1.83 (m)	2.09 (m), 1.82 (m)	2.09 (m), 1.83 (m)	2.17 (m), 1.92 (m)
15	1.94 (m), 1.60 (m)	1.91 (m), 1.61 (m)	1.94 (m), 1.60 (m)	1.91 (m), 1.61 (m)	2.60 (m), 2.04 (m)
16	1.92 (m), 1.87 (m)	1.92 (m)	1.92 (m), 1.87 (m)	1.93 (m)	2.46 (m), 2.08 (m)
17	2.32 (m)	2.34 (m)	2.28 (m)	2.32 (m)	2.95 (t, 9.2)
18	0.85 (s)	0.84 (s)	0.81 (s)	0.85 (s)	1.22 (s)
19	0.95 (s)	0.95 (s)	0.95 (s)	0.96 (s)	1.07 (s)
21	1.13 (s)	1.15 (s)	1.15 (s)	1.15 (s)	1.59 (s)
22	3.65 (dd, 8.8, 3.6)	3.64 (m)	3.68 (dd, 9.6, 2.8)	3.63 (m)	3.86 (d, 10.4)
23	1.49 (m)	1.54 (m)	1.47 (m)	1.52 (m)	1.94 (m), 1.63 (m)
24	1.50 (m), 1.15 (m)	1.72 (m), 1.45 (m)	1.68 (m), 1.15 (m)	1.71 (m)	2.17 (m), 1.41 (m)
25	1.61 (m)		1.63 (m)		1.81 (m)
26	3.34 (dd, 10.4, 6.4)	1.19 (s)	3.34 (dd, 10.4, 6.4)	1.19 (s)	3.64 (dd, 10.0, 6.4)
	3.42 (dd, 10.4, 5.6)		3.42 (dd, 10.4, 5.6)		3.76 (dd, 10.0, 5.2)
27	0.93 (d, 6.8)	1.20 (s)	0.93 (d, 6.4)	1.20 (s)	1.03 (d, 6.4)
1'	5.05 (q, 4.8)	4.97 (t, 4.0)		5.05 (q, 4.8)	
2'	1.29 (d, 4.8)	1.90 (m)	1.30 (s)	1.29 (d, 4.8)	
3'		2.41 (t, 7.2)	1.37 (s)		
4'-OCH_3_		3.65 (s)			

^a^ Data were measured in CD_3_OD; ^b^ Data were measured in C_5_D_5_N.

**Table 2:** The ^13^C-NMR (100 MHz) spectral data [*δ* (ppm)] of compounds **1**–**4** and **8**.

Position	*δ*_C_ (1) ^a^	*δ*_C_ (2) ^a^	*δ*_C_ (3) ^a^	*δ*_C_ (4) ^a^	*δ*_C_ (8) ^b^
1	37.3 (t)	37.3 (t)	37.3 (t)	37.3 (t)	38.0 (t)
2	68.7 (d)	68.7 (d)	68.7 (d)	68.7 (d)	68.1
3	68.5 (d)	68.5 (d)	68.5 (d)	68.5 (d)	68.1
4	32.9 (t)	32.8 (t)	32.8 (t)	32.9 (t)	32.5 (t)
5	51.8 (d)	51.8 (d)	51.8 (d)	51.8 (d)	51.4 (d)
6	206.4 (s)	206.5 (s)	206.4 (s)	206.4 (s)	203.5 (s)
7	122.2 (d)	122.2 (d)	122.2 (d)	122.1 (d)	121.7 (d)
8	167.5 (s)	167.6 (s)	167.6 (s)	167.6 (s)	166.1 (s)
9	35.1 (d)	35.1 (d)	35.1 (d)	35.1 (d)	34.5 (d)
10	39.2 (s)	39.2 (s)	39.2 (s)	39.2 (s)	38.7 (s)
11	21.5 (t)	21.5 (t)	21.5 (t)	21.5 (t)	21.1 (t)
12	32.2 (t)	32.2 (t)	32.3 (t)	32.1 (t)	31.8 (t)
13	49.0 (s) *	49.0 (s) *	49.0 (s) *	49.0 (s) *	48.1 (s)
14	85.2 (s)	85.2 (s)	85.3 (s)	85.2 (s)	84.2 (s)
15	31.7 (t)	31.7 (t)	31.7 (t)	31.7 (t)	32.1 (t)
16	22.6 (t)	22.6 (t)	22.4 (t)	22.6 (t)	21.7 (t)
17	51.3 (d)	51.4 (d)	50.5 (d)	51.3 (d)	50.1 (d)
18	17.6 (q)	17.6 (q)	17.6 (q)	17.6 (q)	17.9 (q)
19	24.4 (q)	24.4 (q)	24.4 (q)	24.4 (q)	24.5 (q)
20	85.3 (s)	85.3 (s)	85.7 (s)	85.3 (s)	77.3 (s)
21	23.6 (q)	23.4 (q)	22.5 (q)	23.7 (q)	21.5 (q)
22	85.5 (d)	85.6 (d)	83.1 (d)	85.6 (d)	76.8 (d)
23	27.3 (t)	24.6 (t)	27.4 (t)	24.6 (t)	30.3 (t)
24	32.0 (t)	42.2 (t)	32.0 (t)	42.2 (t)	32.0 (t)
25	37.0 (d)	71.1 (s)	37.0 (d)	71.1 (s)	36.8 (d)
26	68.2 (t)	29.5 (q)	68.2 (t)	29.5 (q)	67.4 (t)
27	17.0 (q)	28.9 (q)	17.0 (q)	28.9 (q)	17.8 (q)
1'	102.3 (d)	103.9 (d)	108.0 (s)	102.3 (d)	
2'	22.0 (q)	31.0 (t)	29.3 (q)	22.0 (q)	
3'		29.2 (t)	27.2 (q)		
4'		175.6 (s)			
4'-OCH_3_		52.1 (q)			

^a^ Data recorded in CD_3_OD; ^b^ Data recorded in C_5_D_5_N; * The signal is overlapped by solvent.

Compound **2** was also obtained as a white amorphous powder. HR-EIMS showed a [M]^+^ ion at *m/z* 578.3450, corresponding to the molecular formula C_32_H_50_O_9_ (calcd for C_32_H_50_O_9_, 578.3449). The spectroscopic data (IR, UV, and NMR: See Section 3.3. and Table 1 and Table 2) suggested **2** was an ecdysteroid closely related to 20,22-*O*-(*R*-ethylidene)-20-hydroxyecdysone, a reported [24,25] ecdysteroid which was also obtained in the present study (compound **4**). Further comparison of the ^1^H- and ^13^C-NMR spectra showed that the signals for the C_2_ acetal group in **4** were absent in **2**. Instead, additional signals for one acetalated 3-methoxycarbonyl-propylal group [*δ*_H_ 4.97 (1H, t, H-1'), 3.65 (3H, s, 4'-OCH_3_), 2.41 (3H, H_3_-3'), 1.90 (3H, H_3_-2'); *δ*_C_ 175.6 (C-4'), 103.9 (C-1'), 52.1 (4'-OCH_3_), 31.0 (C-3')_,_ 29.2 (C-2')] were exhibited in the spectra. ^1^H-^1^H COSY and HSQC spectra permitted establishment of the spin system from C-1' through C-3'. The HMBC correlations from 4'-OCH_3_ (*δ*_H_ 3.65), H_2_-3' (*δ*_H_ 2.41) and H_2_-2' (*δ*_H_ 1.91) to C-4' (*δ*_C_ 175.6), and from H_2_-2', H_2_-3' to C-1' (*δ*_C_ 103.9) indicated the linkage from C-1' to 4'-OCH_3_. The *R* configuration at C-1' was determined by the important NOE correlation between H-1' and H-22 (Figure 2). These findings led to the establishment of the whole structure of **2** as shown in Figure 1, and this structure was further well supported by other important ^1^H-^1^H COSY, HMBC and NOESY correlations. Therefore, **2** was determined as 20,22-*O*-(*R*-3-methoxycarbonyl)propylidene-20-hydroxyecdysone. 

Compound **3**, showing the molecular formula C_28_H_44_O_7_, was deduced to be the same ecdysteroid compound inokosterone-20,22-acetonide from the roots of *Leuzea carthamoides* recently reported in the literature [22], by comparison of its spectroscopic data (Table 1 and Table 2) with reported values. However, at that time the authors had yet not clarified the absolute configuration at C-25 in the structure. In order to determine the stereochemistry at C-25 in the structure of **3**, a similar acid hydrolysis like that performed for **1** was conducted, and the free ecdysteroid that was released from **3** was confirmed to be (25*S*)-inokosterone (**8**) on the basis of a co-TLC elution test and NMR analyses, suggesting that the absolute configuration at C-25 in **3** should also be *S* configuration. Therefore, the complete structure of **3** was determined as (25*S*)-inokosterone-20,22-acetonide.

The other five known compounds were identified as 20,22-*O*-(*R*-ethylidene)-20-hydroxyecdysone (**4**) [24,25], 20-hydroxyecdysone-20,22-monoacetonide (**5**) [25,26], 20-hydroxyecdysone (**6**) [27], (25*R*)-inokosterone (**7**) [23,28] and (25*S*)-inokosterone (**8**) [23,28], by interpretation of their spectroscopic data, as well as by comparison with literature values.

Among the eight isolated compounds, **1** and **2** are two new phytoecdysteroids, each characterized by having an acetal group in the molecule. In particular, **2** is so far the first example of phytoecdysteroid acetalated at the side chain with a 4-oxobutanoic acid unit. Compounds **3**–**5** were found in *Achyranthes bidentata* roots for the first time.

## 3. Experimental

### 3.1. General

Optical rotations were measured on a Perkin-Elmer 341 polarimeter with MeOH as solvent. UV spectra were recorded in MeOH on a Perkin-Elmer Lambda 35 UV-Vis spectrophotometer. IR spectra (KBr) were taken on a Bruker Tensor 27 spectrophotometer in cm^−1^. NMR spectra were recorded in C_5_D_5_N and CD_3_OD on a Bruker DRX-400 instrument using the residual solvent peak as reference. ESIMS were collected on an MDS SCIEX API 2000 LC/MS/MS instrument. HRESIMS data were obtained on a Water Q-TOF Premier mass spectrometer and HREIMS data were obtained on a Finigan MAT 95XP mass spectrometer. Preparative HPLC was conducted using a CXTH P3000 HPLC pump and a UV3000 UV-Vis Detector with a Fuji-C18 column (10 µm–100 A). For column chromatography (CC), silica gel (200–300 mesh, Qingdao Marine Chemical Inc., Qingdao, China), YMC ODS-A (50 μm, YMC Co. Ltd., Kyoto, Japan) and Sephadex LH-20 (Pharmacia Fine Chemical Co. Ltd., Uppsala, Sweden) were used. Fractions were monitored by TLC, and spots were visualized by heating the silica gel plates sprayed with 10% H_2_SO_4_ in ethanol.

### 3.2. Plant Materials

Roots of *A. bidentata* Blume were purchased from Anguo Professional Market for Chinese Materia Medica, in April 2010, and were collected in Anguo County, Hebei Province, China. Plants were authenticated by Fu-Wu Xing (South China Botanical Garden, Chinese Academy of Sciences), and a voucher specimen (No. 20100408A) was deposited in the Laboratory of Phytochemistry of South China Botanical Garden, Chinese Academy of Sciences.

### 3.3. Extraction and Isolation

Powder of the dry roots of *A. bidentata* (5.10 kg) was extracted with EtOH-H_2_O (95:5, 10 L × 3) at room temperature three times (24 h each). The EtOH extracts were combined and concentrated *in vacuo*. Then, the resulting residue was suspended in H_2_O (1.5 L) and sequentially extracted with petroleum ether (5 L × 3), EtOAc (5 L × 3) and *n*-BuOH (5 L × 3). The EtOAc layer was evaporated *in vacuo* to yield EtOAc-soluble fraction (17.5 g).

The EtOAc-soluble fraction was subjected to silica gel CC using a gradient of CHCl_3_-MeOH (95:5–60:40, v/v) to give ten fractions (E_1_–E_10_). Fraction E_8_ (3.90 g), obtained by elution with CHCl_3_-MeOH (85:15, v/v), was further subjected to silica gel CC and successively eluted with CHCl_3_-MeOH (20:1–10:1, v/v) to yield six sub-fractions (E_6–1_–E_6–6_). Sub-fraction E_6–4_ (0.370 g) was separated by an ODS column using MeOH-H_2_O (60:40–100:0, v/v), followed by HPLC preparation with MeOH-H_2_O (70:30, v/v) at a flow rate of 10 mL/min to afford compounds **2** (5.9 mg, t_R_ = 41 min), **4** (6.8 mg, t_R_ = 49 min), **3** (6.0 mg, t_R_ = 61 min), **1** (5.0 mg, t_R_ = 65 min), and **5** (9.3 mg, t_R_ = 67 min). Sub-fraction E_6–5_ (0.200 g ) was purified by Sephadex LH-20 CC using MeOH as eluent, followed by preparative HPLC using MeOH-H_2_O (40:60, v/v) at a flow rate of 8 mL/min to afford compounds **6** (t_R_ = 96 min, 20.0 mg), **7** (t_R_ = 114 min, 29.0 mg) and **8** (t_R_ = 127 min, 38.0 mg).

*(25S)-20,22-O-(R-Ethylidene)inokosterone* (**1**). White amorphous powder; [α]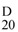 + 26.8 (c = 0.35, MeOH); IR (KBr) *ν*_max_ 3419, 2934, 1654 ,1450, 1139, 1156 cm^−1^; UV (MeOH) *λ*_max_ (log *ε*) nm: 242 (4.14); ESIMS (+) *m*/*z* 529 [M+Na]^+^, 507 [M+H]^+^; ESIMS (−) *m*/*z* 505 [M–H]^–^; HRESIMS (−) *m/z* 505.3173 [M−H]^–^ (calcd. for C_29_H_45_O_7_, 505.3160); ^1^H-NMR (CD_3_OD, 400 MHz) and ^13^C-NMR (CD_3_OD, 100 MHz) data are shown in Table 1 and Table 2.

*20,22-O-(R-3-Methoxycarbonyl)propylidene-20-hydroxyecdysone* (**2**). White amorphous powder; [α]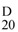 + 34.0 (c = 0.20, MeOH); IR (KBr) *ν*_max_ 3423, 2964, 1737, 1654, 1382, 1139, 1058 cm^−1^; UV (MeOH) *λ*_max_ (log *ε*) nm: 242 (3.75); ESIMS (+) *m*/*z* 601 [M+Na]^+^, 579 [M+H]^+^; ESIMS (−) *m*/*z* 577 [M−H]^−^; HREIMS *m/z* 578.3450 [M]^+^ (calcd. for C_32_H_50_O_9_, 578.3449); ^1^H-NMR (CD_3_OD, 400 MHz) and ^13^C-NMR (CD_3_OD, 100 MHz) data are shown in Table 1 and Table 2.

*(25S)-Inokosterone-20,22-acetonide* (**3**). White amorphous powder; ESIMS (+) *m*/*z* 520 [M+Na]^+^; ESIMS (−) *m*/*z* 555 [M+Cl]^−^, 519 [M−H]^−^; ^1^H-NMR (CD_3_OD, 400 MHz) and ^13^C-NMR (CD_3_OD, 100 MHz) data are shown in Table 1 and Table 2.

*20,22-O-(R-Ethylidene)-20-hydroxyecdysone* (**4**). White amorphous powder; ESIMS (+) *m*/*z* 545 [M+K]^+^, 529 [M+Na]^+^; ESIMS (−) *m*/*z* 541 [M+Cl]^−^, 505 [M−H]^−^; ^1^H-NMR (CD_3_OD, 400 MHz) and ^13^C-NMR (CD_3_OD, 100 MHz) data are shown in Table 1 and Table 2.

*(25S)-Inokosterone* (**8**). White amorphous powder; ESIMS (+) *m*/*z* 519 [M+K]^+^, 503 [M+Na]^+^; ESIMS (−) *m*/*z* 515 [M+Cl]^−^; ^1^H-NMR (C_5_D_5_N, 400 MHz) and ^13^C-NMR (C_5_D_5_N, 100 MHz) data are shown in Table 1 and Table 2.

### 3.4. Acidic Hydrolysis of Compounds 1 and 3

Individual solutions of **1** (2.5 mg) and **3** (3.0 mg) in 1 M H_2_SO_4_ (0.5 mL) were allowed to stand at room temperature for 12 h with continuous oscillation. Each reaction mixture was subsequently extracted with CHCl_3_ (1 mL) and EtOAc (1 mL × 3), and the EtOAc-soluble fraction was concentrated to dryness to give a free ecdysteroid (1.5 mg from **1** and 1.8 mg from **3**). Both hydrolysates were identified as the known compound (25S)-inokosterone (**8**).

## 4. Conclusions

Two new phytoecdysteroids, (25*S*)-20,22-*O*-(*R*-ethylidene)inokosterone (**1**) and 20,22-*O*-(*R*-3-methoxycarbonyl)propylidene-20-hydroxyecdysone (**2**), were isolated from the roots of *Achyranthes bidentata* Blume, along with six known ones. Three out of the six known compounds **3**–**5** were found in this plant species for the first time. Compound **2** is so far the first example of phytoecdysteroid acetalated at the side chain with a 4-oxobutanoic acid unit. The absolute configuration at C-25 in the structure of known compound **3** was further established in this study.

## Supplementary Materials

Supplementary materials can be accessed at: http://www.mdpi.com/1420-3049/17/3/3324/s1.
