# Supplementary material for: Phytoecdysteroids from the Roots of Achyranthes bidentata Blume
Source: Molecules. 2012 Mar 14;17(3):3324–32. doi: 10.3390/molecules17033324 (PMC6268906; doi:10.3390/molecules17033324)

Supplemental Material

In this “Supplement Material” file for the manuscript “Phytoecdysteroids from the Roots of Achyranthes bidentata Blume”, 1D and 2D NMR spectra of new compounds **1–2** are available here as exhibited below.

Contents:

Page 2–7: ^{1}H- and ^{13}C-(DEPT)-NMR, HSQC, HMBC, ^{1}H-^{1}H COSY and NOESY of **1**;
Page 8–13: ^{1}H- and ^{13}C-(DEPT)-NMR, HSQC, HMBC, ^{1}H-^{1}H COSY and NOESY of **2**.

1H-NMR of compound 1.

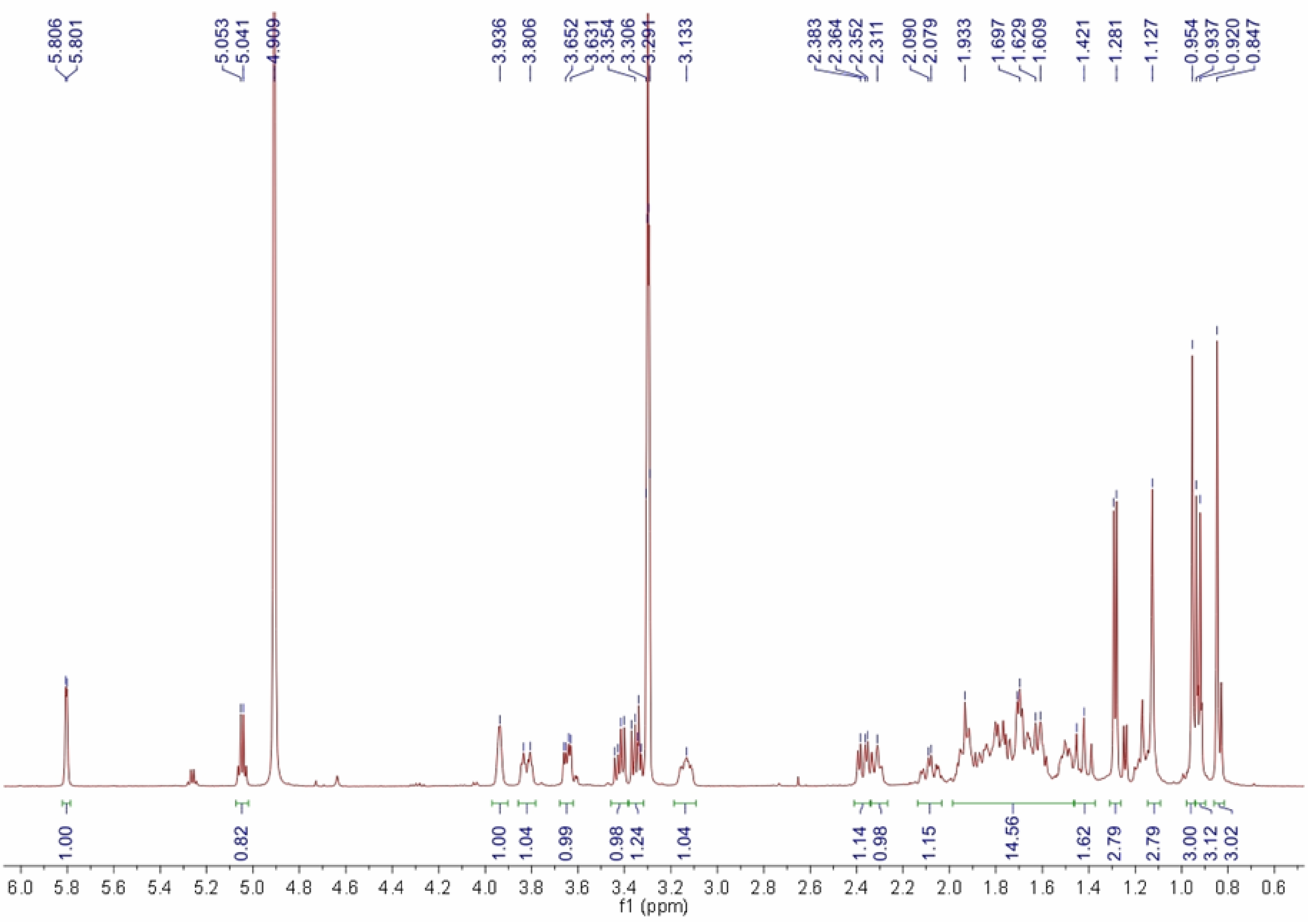

13C (DEPT) NMR of compound **1**.

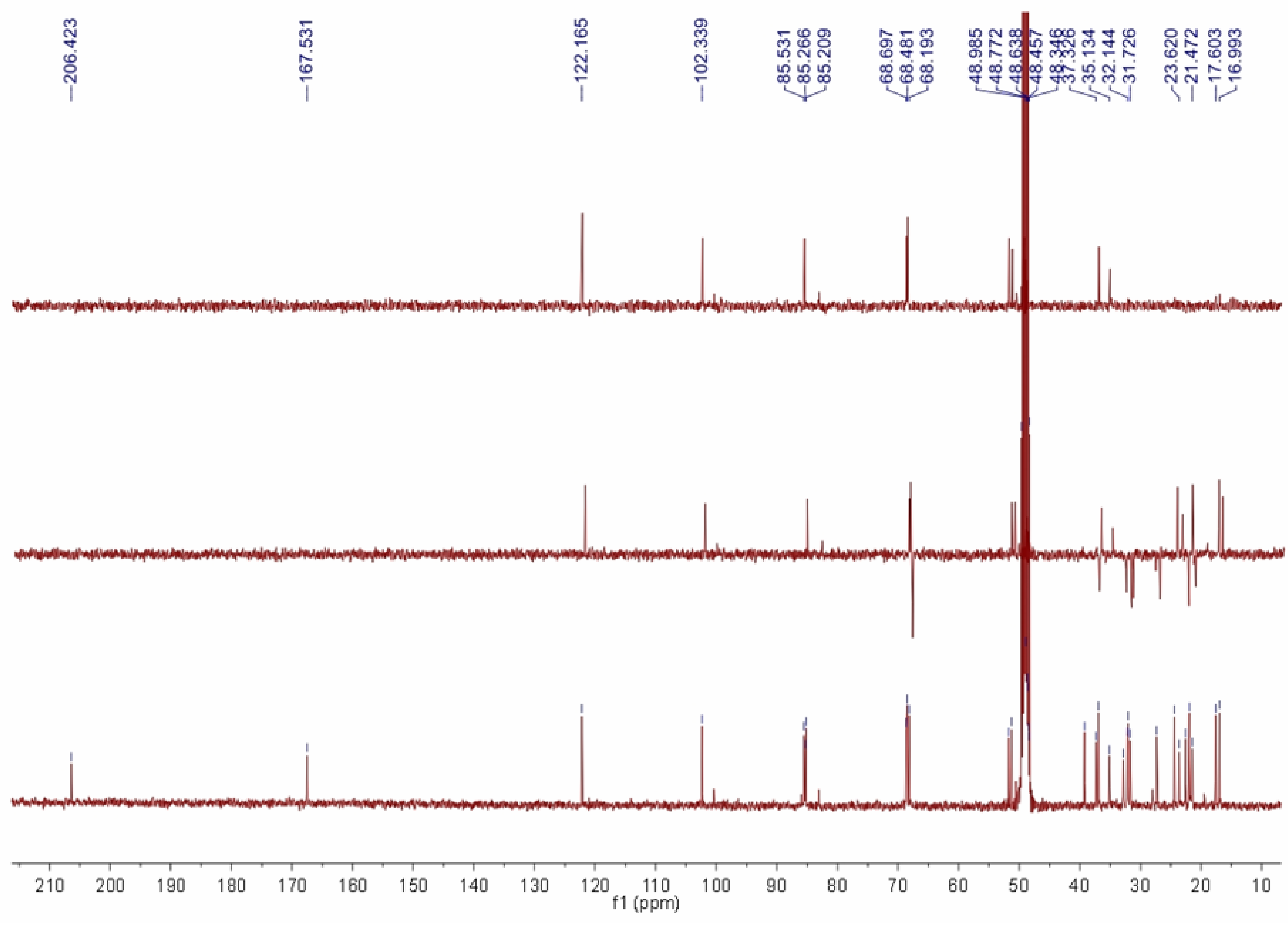

HSQC of compound **1**.

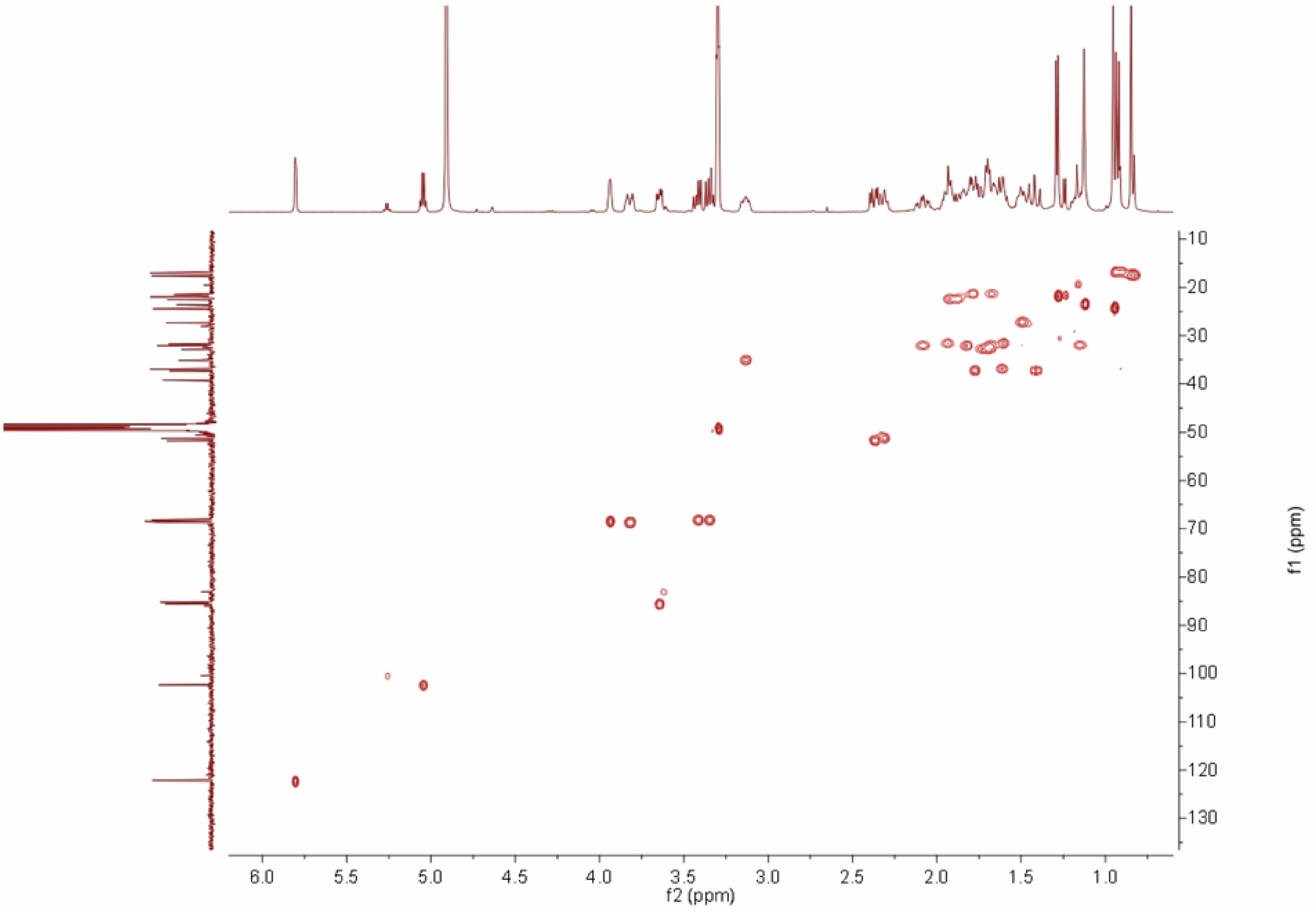

HMBC of compound **1**.

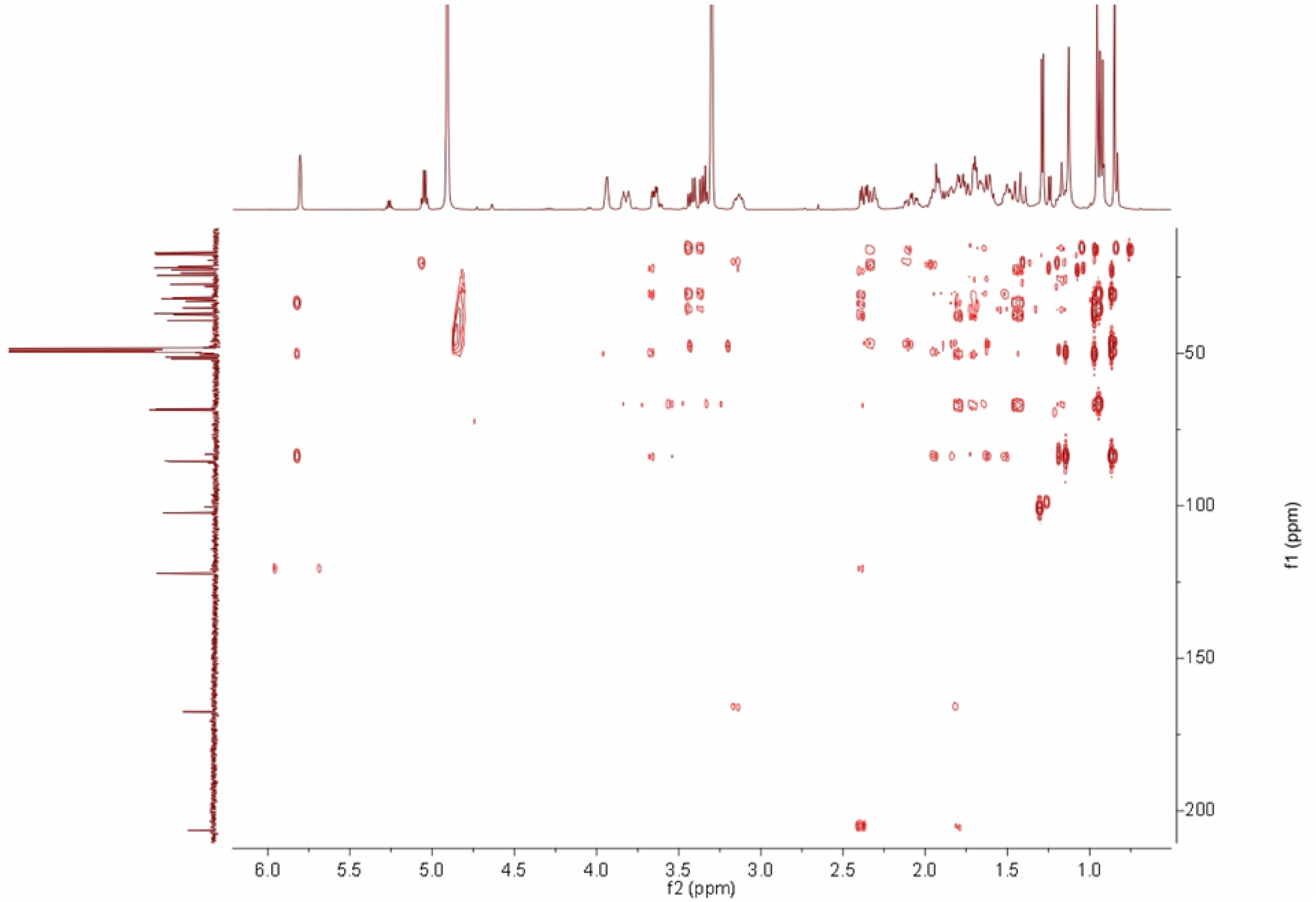

1H-1H COSY of compound **1**.

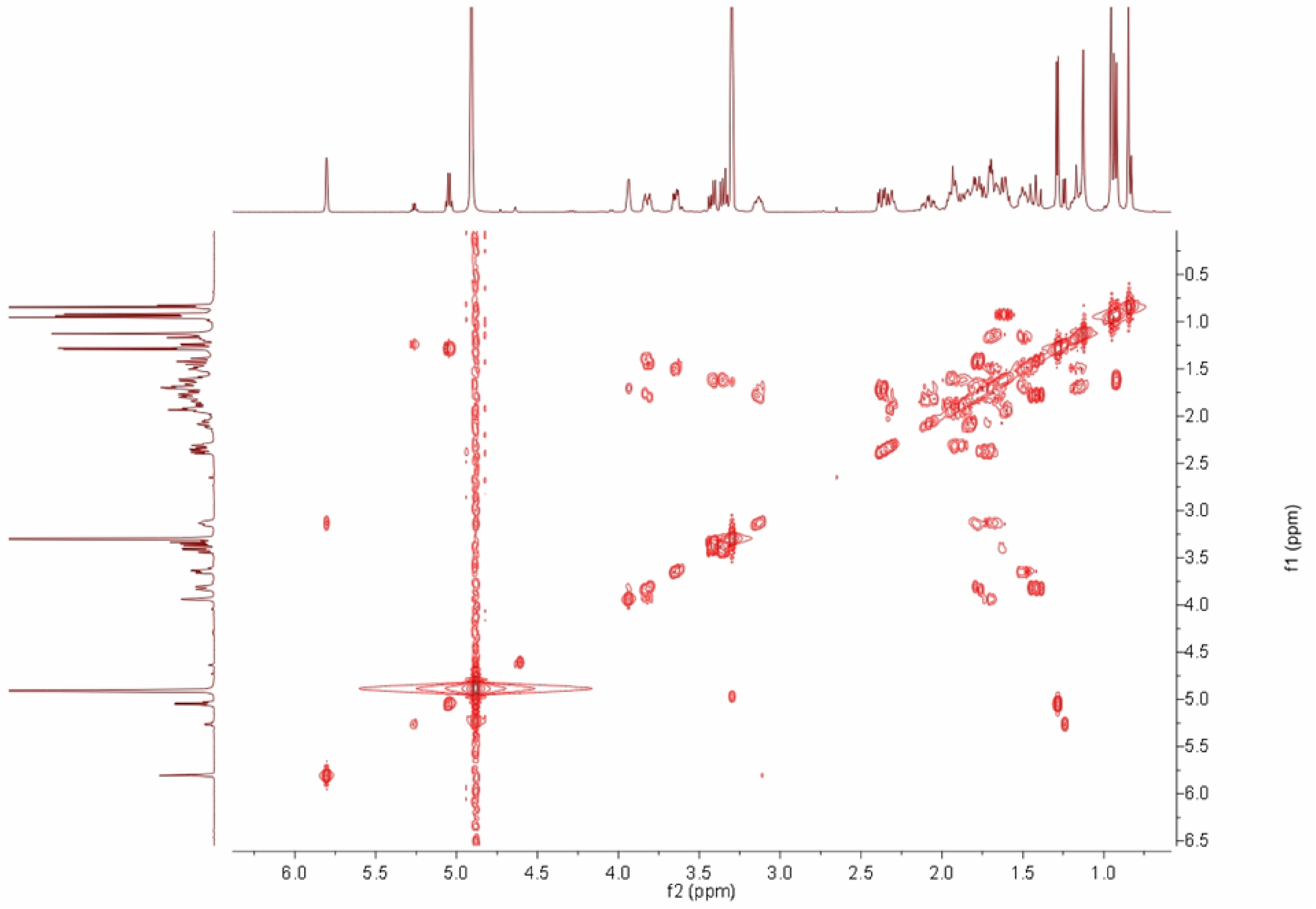

NOESY of compound **1**.

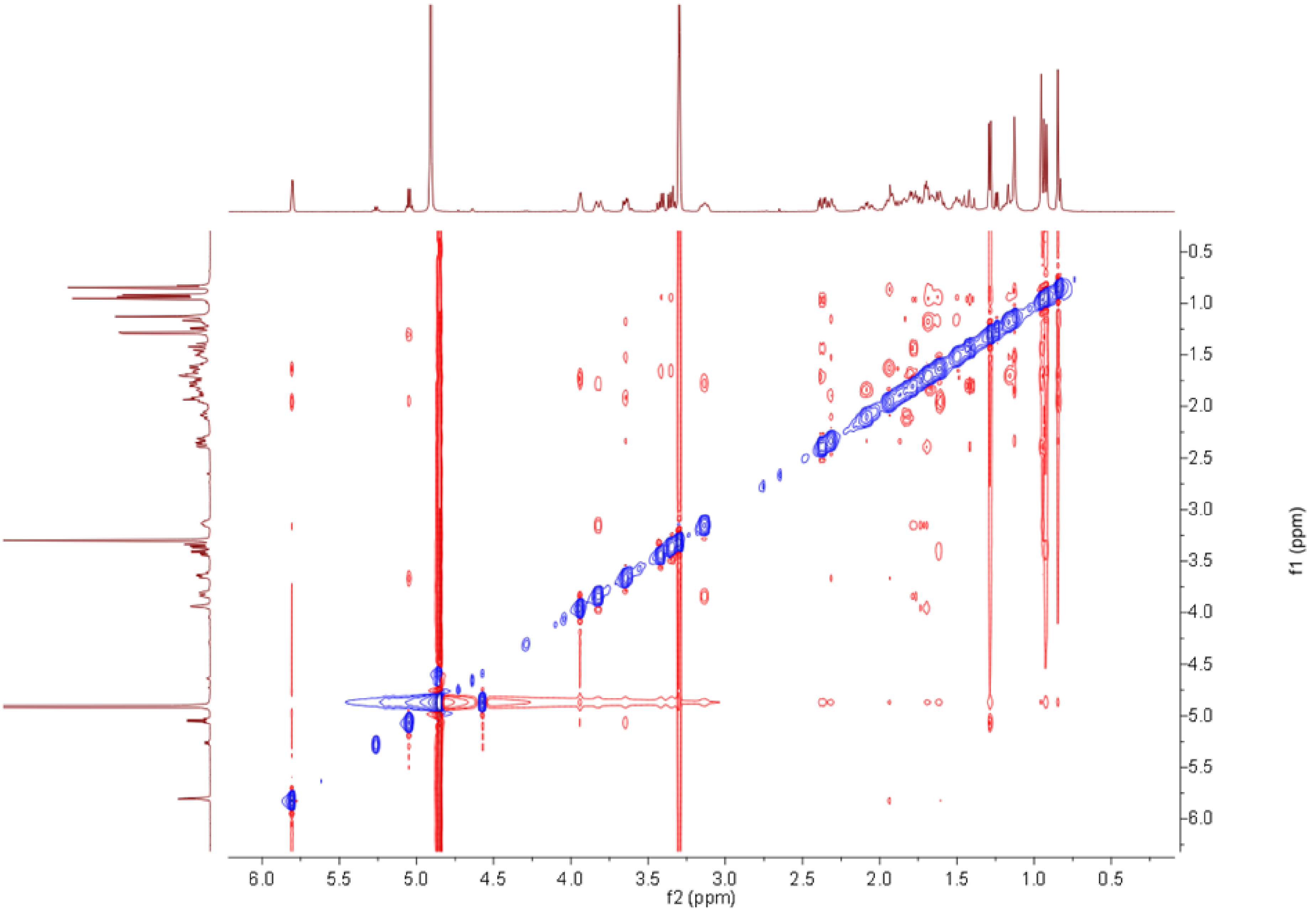

1H-NMR of compound **2**.

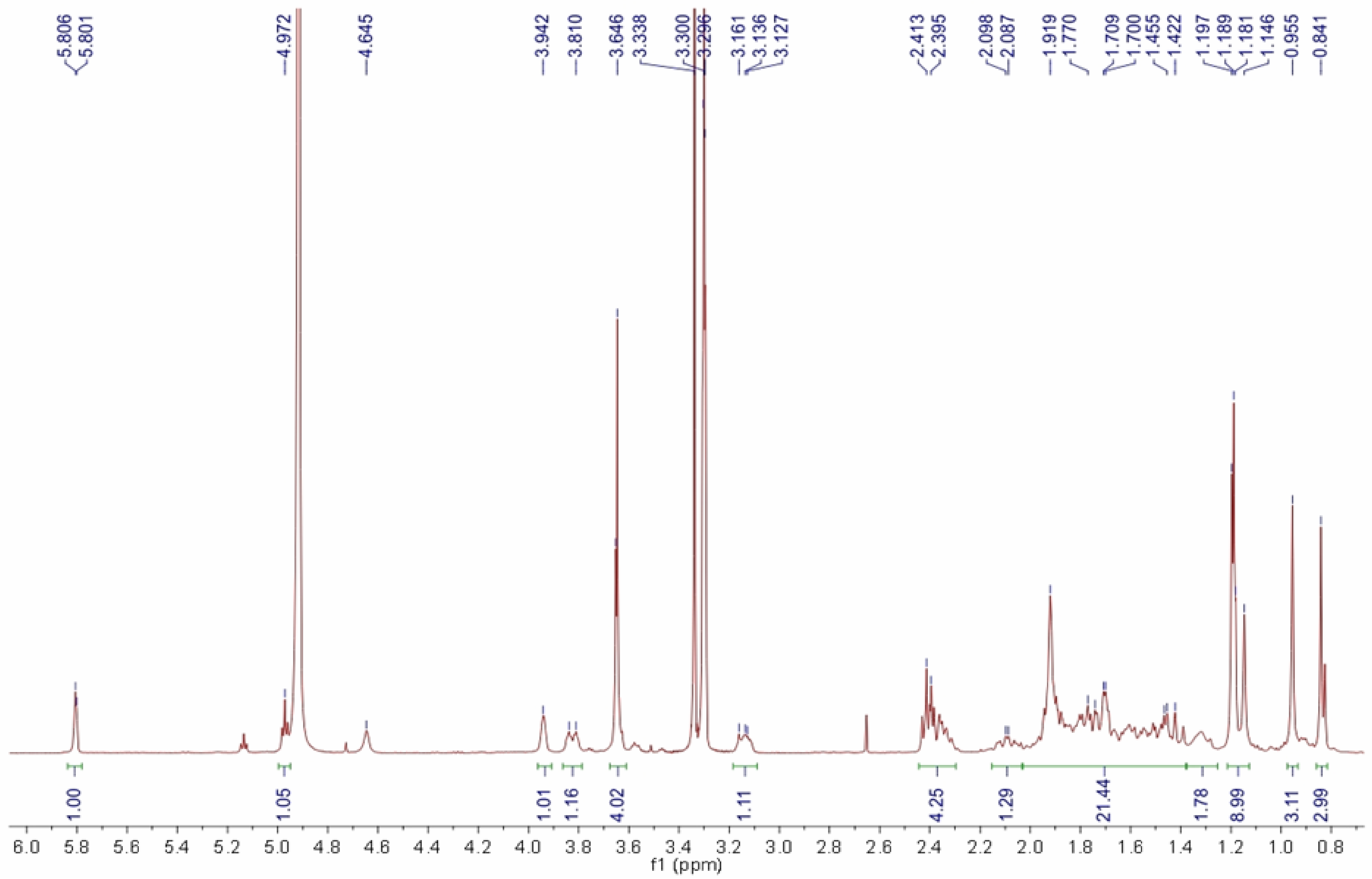

13C (DEPT) NMR of compound **2**.

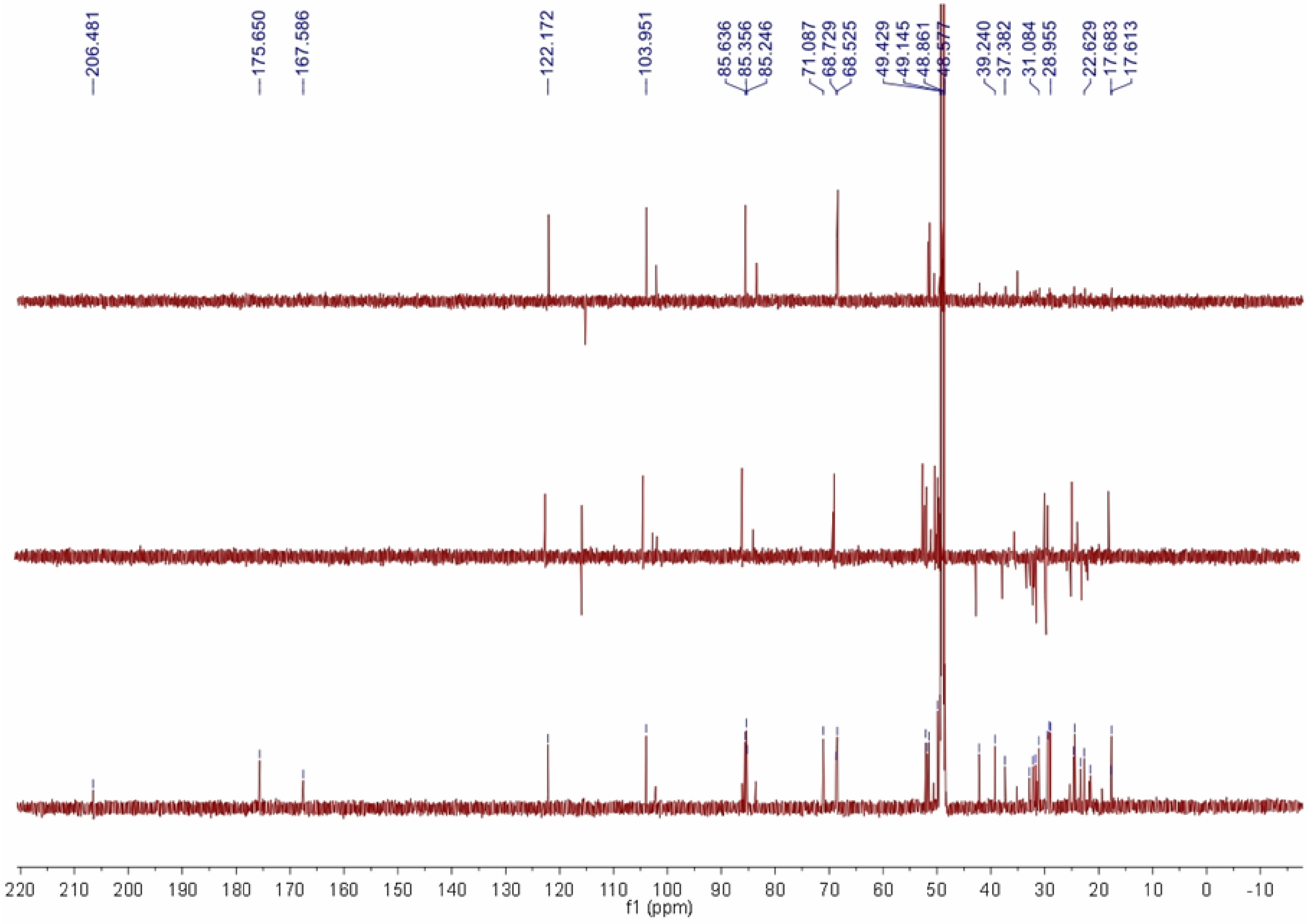

HSQC of compound **2**.

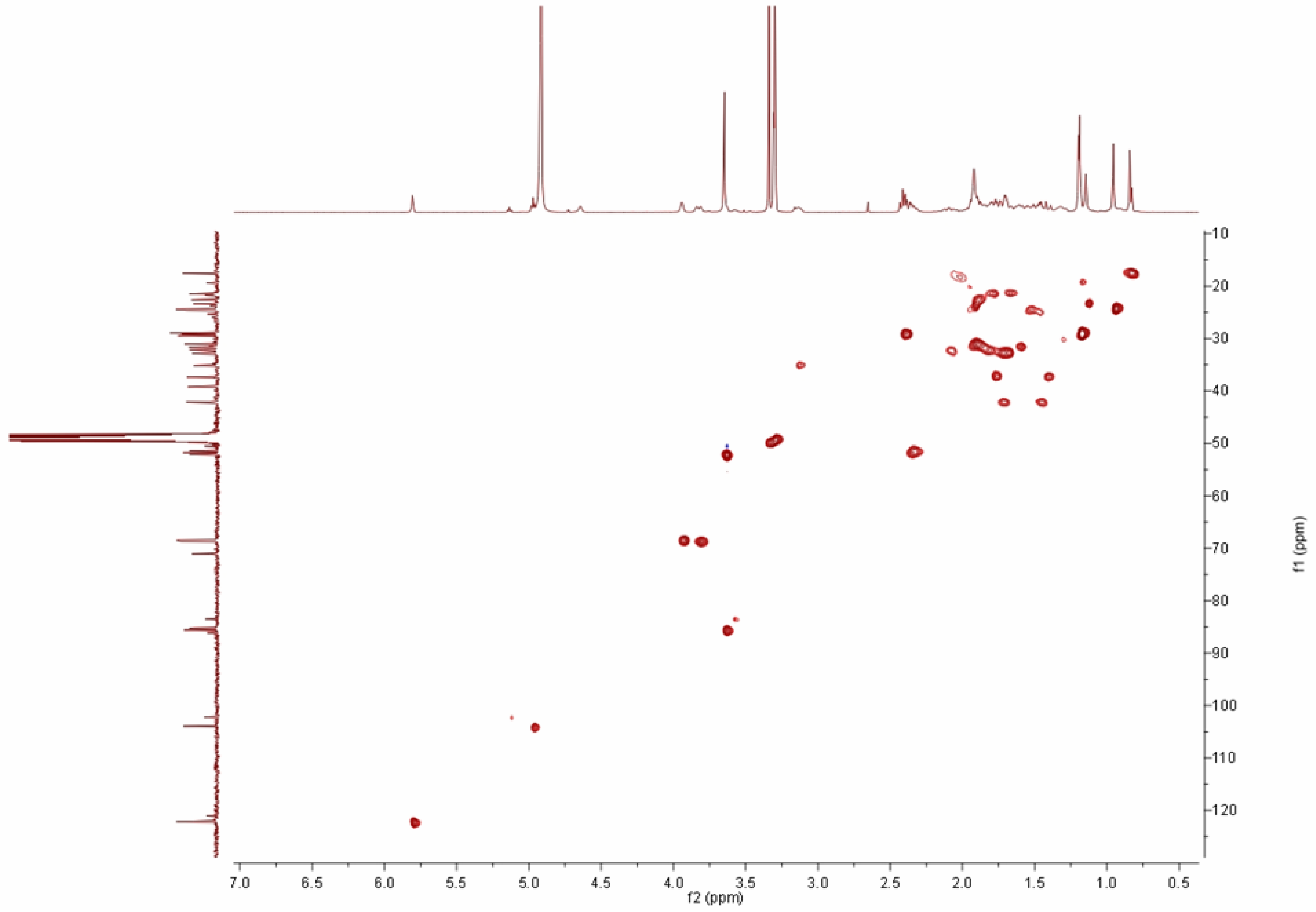

HMBC of compound **2**.

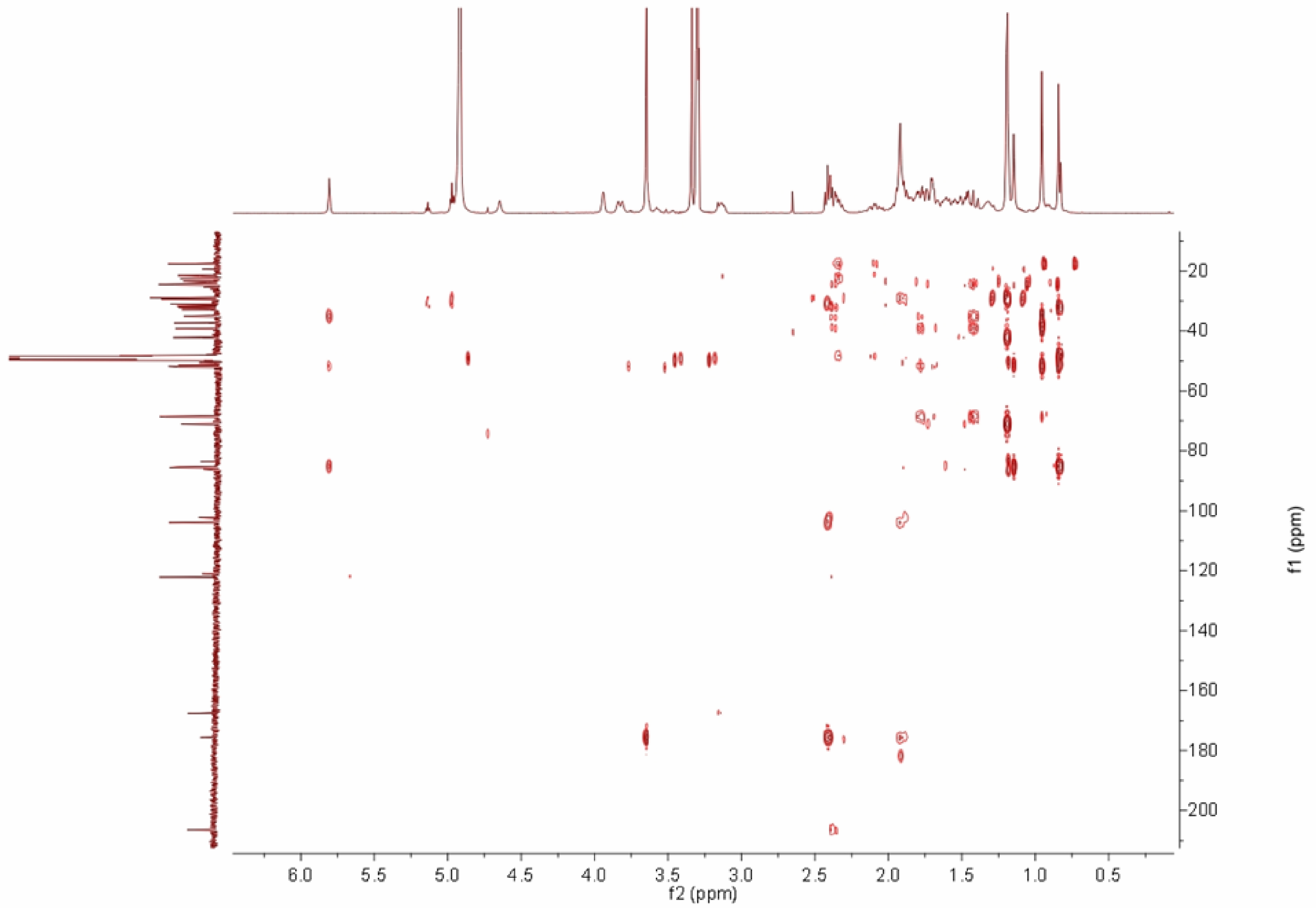

1H-1H COSY of compound **2**.

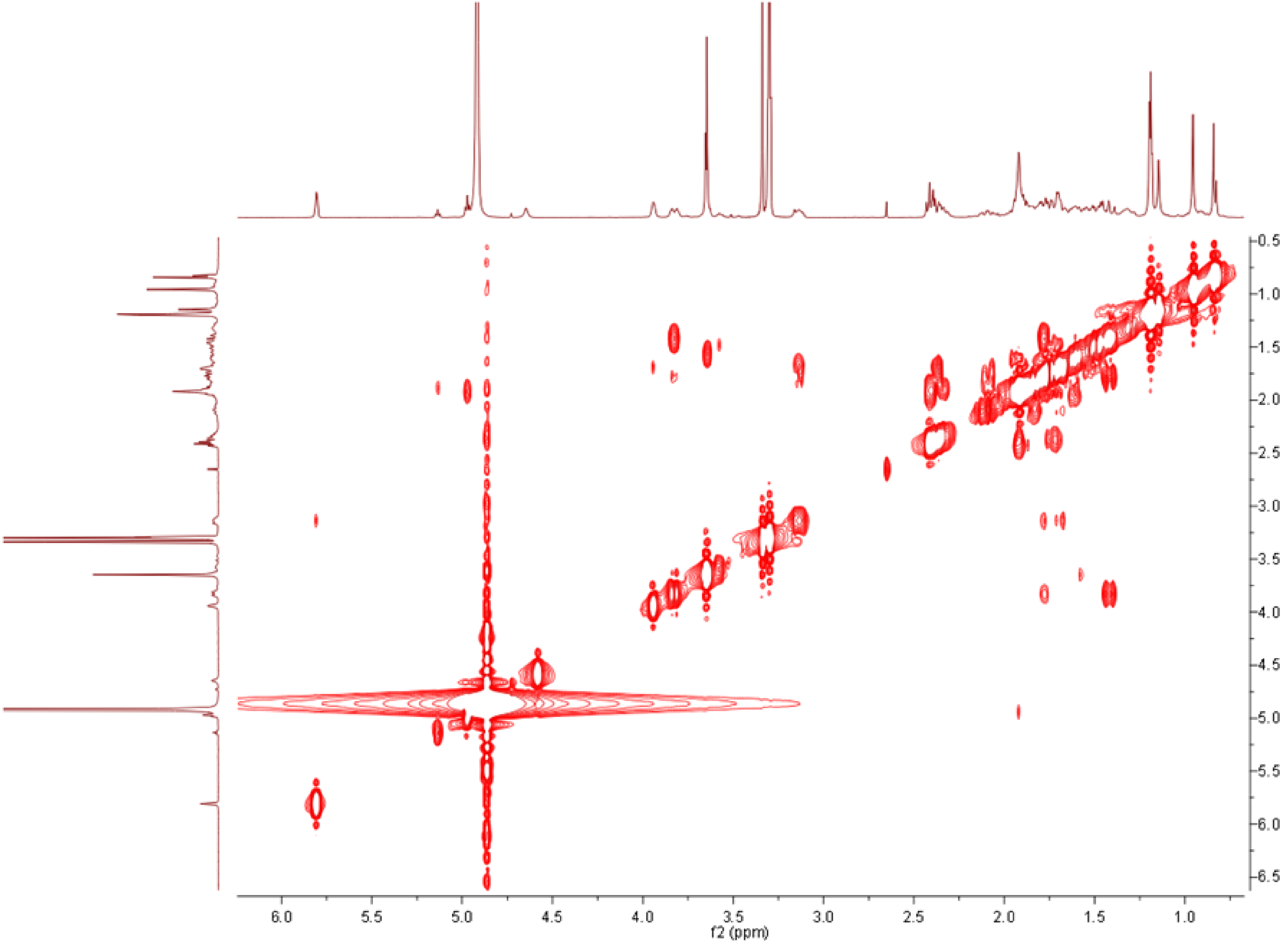

NOESY of compound **2**.

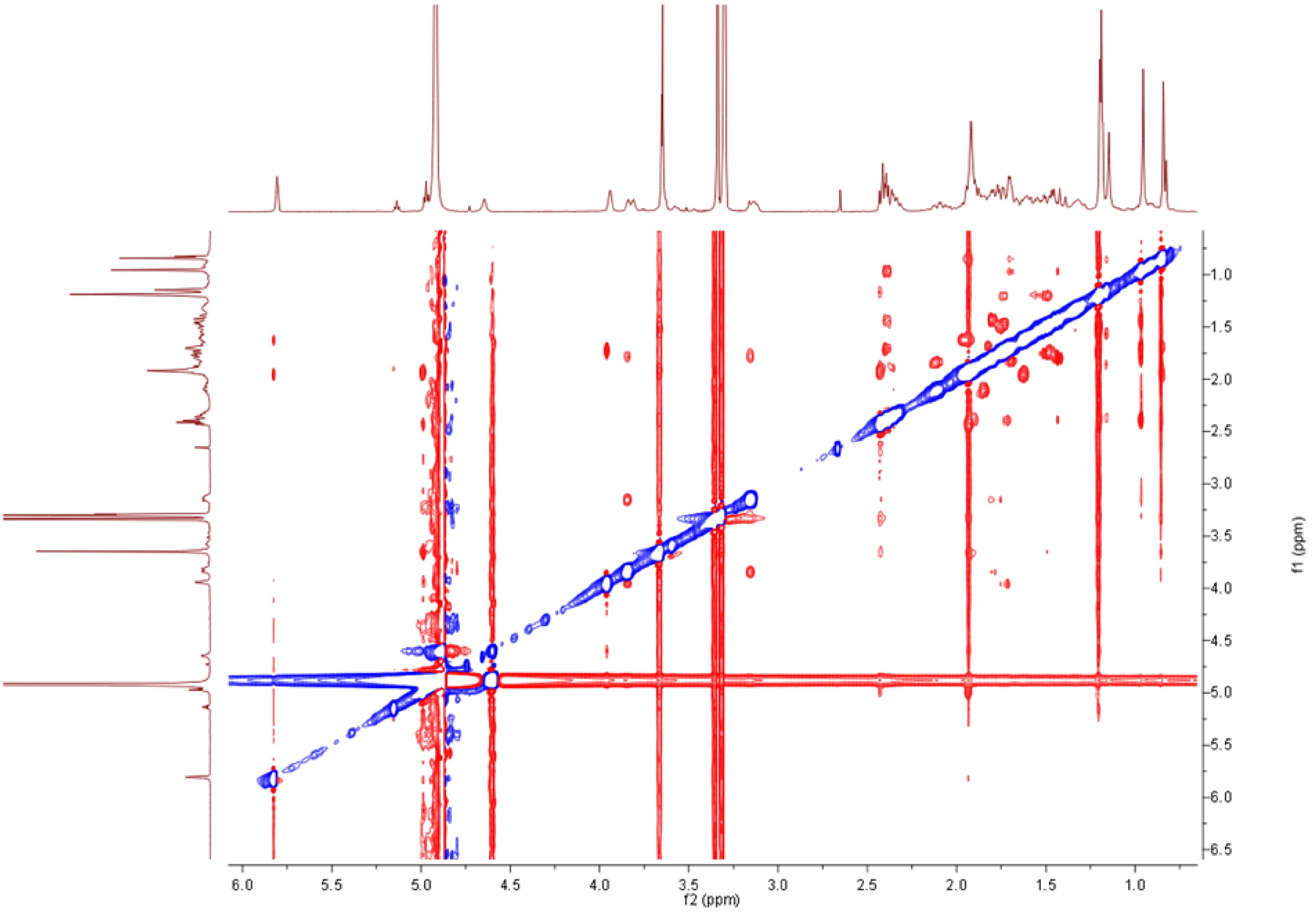

Supplement: Supplementary file 1 [file molecules-17-03324-s001.pdf]
